# Assessing moral competence in medical and psychology students: effects on anxiety and test duration in online versus paper-based testing

**DOI:** 10.1186/s12909-025-08237-w

**Published:** 2025-11-24

**Authors:** Martin Zielina, Tereza Pinkasová, Daniel Dostál, Kateřina Ivanová, Martin Macháček, Jaromír Matějek, Barbora Řebíková, Jakub Kuchař, Jaromír Škoda, Kristýna Pospíšilová, Romana Marková Volejníčková, Adam Doležal

**Affiliations:** 1 Second Faculty of Medicine, Charles University, Prague, Czech Republic; 2 Third Faculty of Medicine, Charles University, Prague, Czech Republic; 3 Faculty of Arts, Palacký University Olomouc, Olomouc, Czech Republic; 4 Faculty of Medicine and Dentistry, Palacký University Olomouc, Olomouc, Czech Republic; 5 Faculty of Economics, VSB-Technical University of Ostrava, Ostrava, Czech Republic; 6 First Faculty of Medicine, Charles University, Prague, Czech Republic; 7 Faculty of Arts, Charles University, Prague, Czech Republic; 8 Institute of Sociology, Czech Academy of Sciences, Prague, Czech Republic; 9 AMBIS University, Prague, Czech Republic

**Keywords:** Moral competence, Anxiety, Medical students, Psychology students, Online vs. paper-based testing

## Abstract

**Background:**

Moral competence and anxiety are essential factors in medical and psychology education, but evidence on how these variables interact across different testing conditions is limited. The present study examined whether moral competence differs between medical and psychology students, how it relates to anxiety levels, and whether test format and duration influence outcomes.

**Methods:**

A total of 717 students (620 medical, 97 psychology) completed the Moral Competence Test (MCT) and the State-Trait Anxiety Inventory (STAI). Participants were systematically (quasi-randomly) assigned to an online or paper-based version of the tests. Test duration was recorded in both formats. Group differences were analyzed using generalized linear models, with additional attention to the relationship between completion time and moral competence.

**Results:**

Psychology students scored higher in moral competence than medical students, while anxiety remained elevated among medical students. Female students reported significantly higher trait anxiety than male students. No significant differences were found between online and paper-based formats in moral competence or anxiety outcomes. Longer test duration was associated with higher moral competence among medical students, although this relationship was correlational and should be interpreted cautiously.

**Conclusions:**

Findings confirm that moral competence declines during medical education while anxiety persists at a higher level, particularly among female students. In contrast, psychology students demonstrated stable or higher moral competence. The absence of differences between online and paper-based formats suggests that both are suitable for assessing moral competence and anxiety in academic settings. The observed association between test duration and moral competence highlights a potential area for further research but should not be interpreted causally.

## Introduction

Healthcare studies are highly demanding and frequently associated with psychological distress, including depression, anxiety, burnout, and related mental health problems [1]. Anxiety in particular has been shown to negatively affect academic performance, decision-making, and students’ overall well-being [2, 3]. Medical students often report higher levels of stress and anxiety than peers in other disciplines, and these symptoms may persist throughout training [1, 4]. They are exposed to constant performance pressure, very frequent and demanding assessments, as well as emotionally challenging clinical situations [5–7]. According to a meta-analysis by Tian-Ci Quek et al. [7], medical students experience anxiety, with possible fluctuation throughout their studies. Some studies suggest that anxiety is highest in the first years because of academic workload and adaptation to stress [8–10], while other studies prove that clinical training can sustain or even heighten anxiety in final years [6, 7]. Psychology students also experience heightened anxiety during their studies, although this issue has not yet been explored as extensively as it has been in medical students [11, 12].

Moral competence represents another essential component of professional development, shaping ethical reasoning and guiding responsible behavior in complex clinical and social situations [13]. Prior studies suggest that medical students may experience a decline in moral competence during their education [14–19], whereas psychology students may benefit from reflective and dialogical practices that support the development of moral reasoning [17, 20, 21]. Among the most frequently cited reasons are a hidden curriculum [15], a decrease in empathy and an increase in cynicism [22], or a loss of ideals from the beginning of study [14]. Despite these findings, empirical comparisons of moral competence and anxiety between medical and psychology students remain scarce. Validated instruments are available to assess these constructs. The State-Trait Anxiety Inventory (STAI) is one of the most widely used measures of state and trait anxiety [3], while the Moral Competence Test (MCT) is a validated tool for evaluating the structure and consistency of moral reasoning [13].

Healthcare studies are frequently linked to anxiety; however, the relationship between anxiety and moral competence remains insufficiently explored. According to Olson [23], state anxiety is negatively associated with moral integrity. Across six studies, Kouchaki and Desai [24] show that anxiety, both induced and measured, can lead to self-interested unethical behavior. On the other hand, there is a study which claims that anxiety has no effect on moral judgment [25]. Empirical research suggests that decision speed can influence moral judgments. Under time pressure, individuals often make more deontological choices—prioritizing moral rules—whereas deliberation tends to increase utilitarian judgments aimed at maximizing the greater good [26, 27]. However, one large-scale study found no systematic difference in moral decisions between time pressure and reflective conditions, suggesting that the contrast between intuitive and deliberative processing may not always affect moral outcomes [28].

Anxiety levels as well as moral competence can vary depending on the type of testing. Some studies indicate that online testing can present challenges related to accessibility, connectivity, and technical issues [29], but the majority of studies suggest that in-class testing is more anxiety-inducing than online, primarily due to the physical presence of examiners and peers [30–32]. However, Sahlan et al. [33] did not find any significant difference in perceived anxiety between online and paper-based testing. Findings from several studies have shown that while certain differences in moral judgment between online and offline environments do exist, these differences tend to be highly context-specific and generally limited in frequency and extent [34].

This study offers a novel contribution by simultaneously examining anxiety and moral competence in both medical and psychology students, two groups that are rarely compared in this context. It further explores whether the mode of test administration (online vs. paper-based) and test completion time are related to levels of anxiety and moral competence, aspects that have not been systematically investigated so far. By addressing these research gaps, the study provides new insights with practical implications for assessment practices in higher education.

The following research questions were examined:

### RQ1

How do anxiety levels differ between first-year and fifth-year medical and psychology students?

### RQ2

How does moral competence differ between first-year and fifth-year medical and psychology students?

### RQ3

What is the relationship between the time required to complete the Moral Competence Test, the level of moral competence, and the level of anxiety?

### RQ4

How do anxiety and moral competence differ depending on whether students complete the test online or on paper?

## Methods

### Participants

Participants were undergraduate students of medicine and psychology recruited from Charles University in Prague and Palacký University in Olomouc, Czech Republic. Data collection took place between January and May 2024. In total, *N* = 717 students participated: three medical faculties (First Faculty of Medicine, *n* = 114; Second Faculty of Medicine, *n* = 191; Third Faculty of Medicine, *n* = 130) and the Department of Psychology, Faculty of Arts (*n* = 53) of Charles University, and one medical faculty (Faculty of Medicine and Dentistry, *n* = 185) and the Department of Psychology, Faculty of Arts (*n* = 44) of Palacký University Olomouc. First-year and fifth-year students were included to capture potential differences between the beginning and final stages of their studies.

Due to incomplete responses in part of the test battery, data on anxiety levels were missing for 65 participants. Consequently, analyses pertaining to anxiety were conducted on a reduced sample of 652 individuals, while all other computations were performed on the complete research sample.

First- and fifth-year students were systematically (quasi-randomly) assigned to either online or paper-based testing conditions, based on study groups or alphabetical order, in agreement with institutional representatives. This procedure ensured an approximately balanced distribution between formats across faculties and years. Some students completed the questionnaires (MCT, STAI, and a short sociodemographic questionnaire) in class on paper, while others were invited to complete them online. Online participants received reminders after 14 days and again after 7 days. At the First Faculty of Medicine, all students completed the questionnaires online, following an institutional decision.

Additional sociodemographic characteristics of the sample are presented in Table 1 in the *Results* section.

### Measures

#### Sociodemographic questionnaire

A short sociodemographic questionnaire collected information regarding year of study (first, fifth), institution (see above), gender (male, female), self-rated religiosity (4-point ordinal scale), test completion duration (in minutes), and self-rated test difficulty (8-point ordinal scale). In test online platform, test completion time was automatically recorded. In the paper-based administration, students were instructed beforehand to report the total time needed, which was then verified by supervising instructors. These characteristics are summarized in Table 1 in the *Results* section.

#### State-trait anxiety inventory (STAI)

Anxiety was assessed using the State-Trait Anxiety Inventory (STAI) [3]. The STAI consists of two subscales, each with 20 items, measuring state anxiety (STAI-X1; temporary, situation-specific) and trait anxiety (STAI-X2; general, dispositional). Items are self-rated on a 4-point Likert scale ranging from “Not at all” to “Very much so.” Subscale scores range from 20 to 80, with higher scores indicating higher anxiety levels. The STAI has been widely used in both research and clinical contexts, with good internal consistency (Cronbach’s alpha > 0.85 for both subscales) and well-established validity across different populations. For the purposes of the present analyses, only the trait subscale (STAI-X2) was used.

#### Moral competence test (MCT)

Moral competence was measured with the Moral Competence Test (MCT) [13]. The MCT presents two moral dilemmas (a Worker’s dilemma and a Doctor’s dilemma), each followed by 12 arguments (six pro and six contra) for each. Respondents rate their level of agreement with each argument on a 9-point Likert scale ranging from “−4 = strongly reject” to “+4 = strongly accept.” The main outcome is the C-score, which ranges from 1 to 100 and reflects the degree of consistency in applying moral principles across different arguments. Higher scores indicate greater moral competence. According to Lind’s categorization [13], scores below 5 indicate no moral competence, 5–9.9 very low, 10–19.9 low, 20–29.9 sufficient, and 30 and above high to very high moral competence. Lind also emphasizes that a score above 20 is desirable for functioning in a free society without the need for external control. Importantly, the C-score does not depend on the content of responses (whether pro or contra), but on the structural consistency of reasoning. The MCT has demonstrated satisfactory reliability and validity in multiple cross-cultural studies.

### Ethical considerations

Participation in the study was voluntary and anonymous. All participants provided informed consent prior to data collection. In the online environment, the informed consent form was presented separately from the questionnaires. The study was conducted in accordance with the Declaration of Helsinki and approved by the ethics committee of the General University Hospital in Prague (162/23 S-IV).

### Statistical analysis

Data were analyzed using mixed-effects regression models. All models included the following predictors: study field (psychology or medicine), year of study (first or fifth), data collection method (online or paper-based), gender (male or female), religiosity (measured on a four-level ordinal scale, treated as a continuous covariate), and age (continuous covariate). Because the data were collected from six different institutions, a random effect for each institution was included. For specific research questions, this common specification was extended with additional predictors and interactions (e.g., test completion time, trait anxiety, and their interactions with field); the exact model for each analysis is described alongside the corresponding results and figures.

Separate statistical models were constructed for two dependent variables: trait anxiety (STAI-X2 raw score) and moral competence (MCT C-score). Link functions were chosen to reflect their distributional and mathematical properties: an identity link (normal regression) for STAI-X2, and a square-root link for the MCT C-score. The square-root link was chosen because the C-score is computed as a sum-of-squares type statistic, and thus inherently reflects a squared quantity. Applying the square-root transformation brings the scale of the dependent variable back in line with the underlying linear component, while also providing a reasonable approximation to normality.

To assure model adequacy, residual diagnostics were conducted. For all models, standardized residuals fell within ± 3 SD, and residual skewness was small (|skew| < 0.25). Visual inspection of QQ-plots confirmed approximate normality, and no major deviations from model assumptions were detected. In addition, marginal *R*^*2*^ values were computed to quantify the proportion of outcome variance explained by the fixed effects alone. Missing data on trait anxiety (27 cases) and test completion time (39 cases) were handled using listwise deletion within the relevant analyses, which reduced the effective sample sizes accordingly; missingness analyses found no evidence of systematic patterns.

Effect sizes (d) were expressed as the difference between the estimated marginal means for the compared groups, divided by the square root of the sum of the residual variance and the variance of the random factor [35]. This definition is directly analogous to Cohen’s d, extended to the context of mixed-effects regression by incorporating both residual and random-effect variance into the denominator. As with the conventional Cohen’s *d*, values of approximately 0.2, 0.5, and 0.8 are typically interpreted as small, medium, and large effects, respectively.

All analyses were conducted in R (version 4.5.1) using the lme4 (version 1.1–37) package for mixed-effects modeling [36] and emmeans (version 1.11.1) for estimated marginal means.

## Results

The study sample consisted of 717 students, including 620 medical students (86%) and 97 psychology students (14%). The majority were women (64%), with men comprising 36%. Most participants were in their first year of study (68%), while 32% were in their fifth year. Nearly half of the participants (48%) were aged 20–21 years. More than one-third of the questionnaires (37%) were completed online, while 63% were administered in paper-based format. A detailed overview of the sociodemographic characteristics is presented in Table 1.

**Table 1:** Demographic characteristics of medical and psychology students

Variable	Count	%
*Gender*		
Women	460	64%
Men	257	36%
*Study field*		
Medicine	620	86%
Psychology	97	14%
*Year of study*		
First year	490	68%
Fifth year	227	32%
*Data collection method*		
Online	264	37%
Paper-based	453	63%
*Age*		
18–19	112	16%
20–21	346	48%
22–23	79	11%
24–25	150	21%
26–30	28	4%
Not stated	2	< 1%
*Religiosity*		
Yes	122	17%
Rather yes	144	20%
Rather no	193	27%
No	258	36%

Notes. Age = years at time of participation. “Year of studies” refers to first- or fifth-year enrollment. *Religiosity* was assessed on a 4-point ordinal scale (1 = strongly religious, 4 = not at all religious). Percentages are based on the total sample (*N* = 717) and may not sum to 100% due to rounding

In Table 2, we present the descriptive statistics for the entire sample for each of the instruments used (MCT, STAI). For the MCT measuring moral competence, we report the total score (C-Score) along with the subscores for the Workers’ and Doctor’s dilemmas, as well as the test completion time. The Workers’ and Doctor’s dilemma subscores showed differential results. While the Workers’ dilemma produced relatively balanced scores, the Doctor’s dilemma yielded noticeably lower C-scores among medical students, suggesting that dilemmas closer to clinical reality (such as euthanasia) may pose a greater challenge to moral reasoning more strongly. These subscores complement the overall C-score by highlighting context-specific variation in moral competence. These subscale differences are consistent with the broader pattern of moral segmentation observed in medical students, where dilemmas tied closely to professional practice evoke lower structural consistency in moral reasoning. For the STAI, we report state anxiety (X1) and trait anxiety (X2) for the entire sample.

**Table 2:** Descriptive statistics of MCT scores, completion times, and STAI

Variable	Mean	St. dev.	Skewness	Five-number summary	Cronbach’s alpha
*Moral Competence Test (MCT)*					
C-Score	25.72	15.12	0.59	0–14–23–36–77	
C-Score (Workers’ dilemma)	43.60	20.61	0.06	0–28–44–59–97	
C-Score (Doctor’s dilemma)	38.90	22.35	0.39	0–21–36–54–97	
Test completion time (minutes)	10.18	4.50	1.97	2–7 – 10–12–40	
*State Trait Anxiety Inventory (STAI)*					
State Anxiety (X1)	47.37	10.55	0.15	23–39–47–55–77	0.92
Trait Anxiety (X2)	45.41	10.09	0.18	21–38–44–53–79	0.91

Notes. Values are means, standard deviations (SD), skewness, and five-number summaries (minimum, 25th percentile, median, 75th percentile, maximum)

Abbreviations. *MCT *Moral Competence Test, *C-Score* Moral Competence Test score, index of moral competence (1–100), *STAI* State-Trait Anxiety Inventory, *X1* State Anxiety subscale, *X2 *Trait Anxiety subscale

**Fig. 1:**
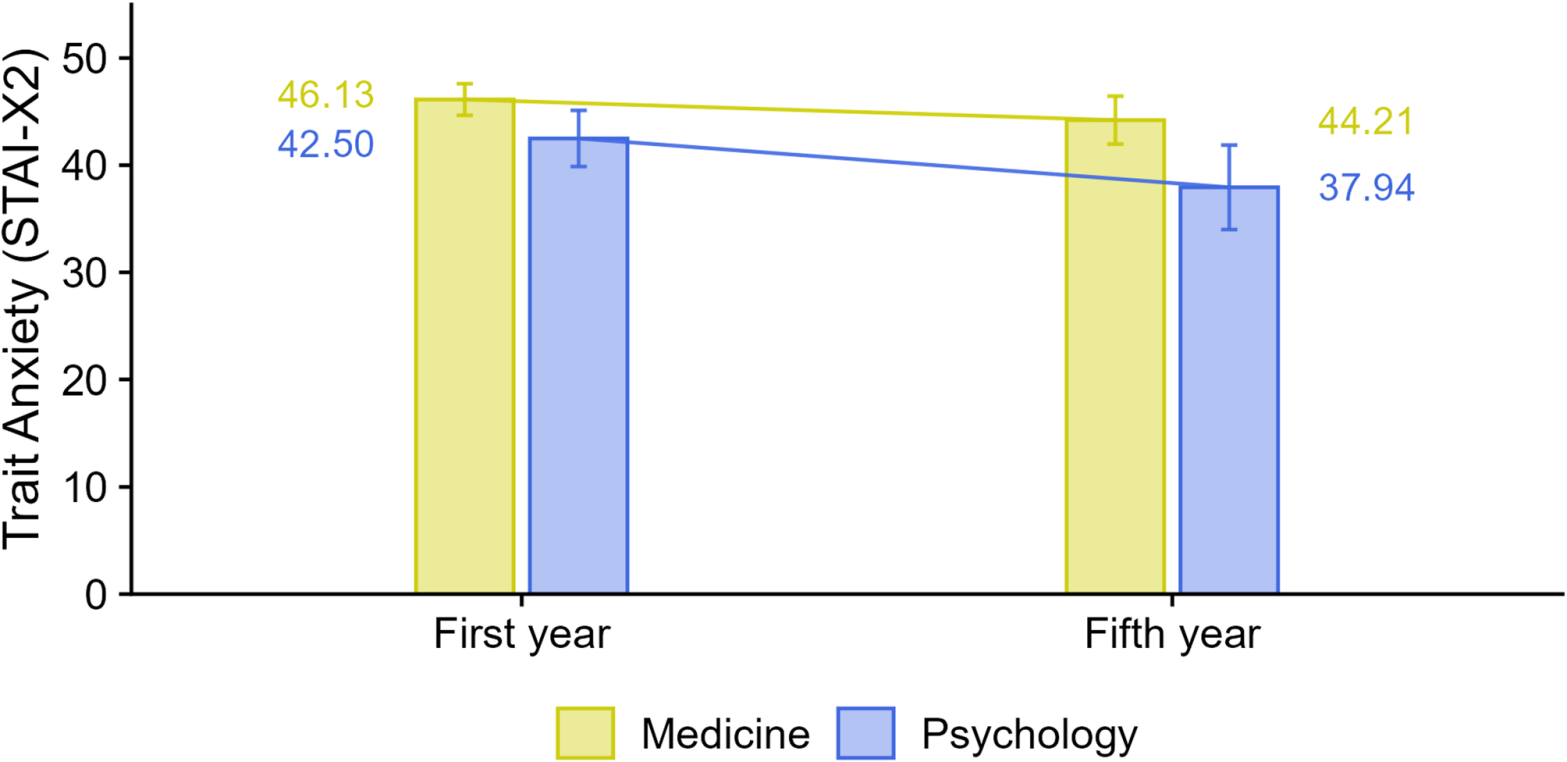
Decreasing level of trait anxiety in fifth year of psychology students. Note. Outcome = STAI-X2 raw score (20–80). Points show estimated marginal means (EMMs) from the mixed-effects model; whiskers = 95% confidence intervals. Groups: Medicine vs. Psychology; First year vs. Fifth year. Sample. Analyses for anxiety used n = 690, collected across six institutions. Key tests. Psychology < Medicine in both years; the between-field difference was larger in the fifth year. Field × Year interaction: t(676.84) = 1.15, p = .249 (not significant). Model fit. The marginal R2 was 0.083, indicating that the fixed effects accounted for about 8% of the variance. Abbreviations. STAI-X2 = Trait Anxiety subscale of the State-Trait Anxiety Inventory

The results (RQ1) confirmed differences in trait anxiety among students based on both field of study and academic phase (i.e., first and fifth year). Specifically, psychology students exhibited lower levels of anxiety compared to medical students. Moreover, this difference tended to increase over the course of the study: in the first year, the difference was approximately 0.37 standard deviations (small to medium effect), t(21.80) = 2.68, *p* = 0.014, whereas in the fifth year, the difference was almost doubled (d = 0.64, medium to large effect; t(78.91) = 3.25, *p* = 0.002). However, the interaction test did not yield statistically significant results, t(676.84) = 1.15, *p* = 0.249, suggesting that the finding of differential development of trait anxiety by field of study remains inconclusive (see Fig. 1).

In addition to the effects of study field and time, a significant effect in trait anxiety was observed only for the gender predictor, with women scoring approximately 0.54 standard deviations higher than men (medium effect), t(682.00) = 6.45, *p* < 0.001.

**Fig. 2:**
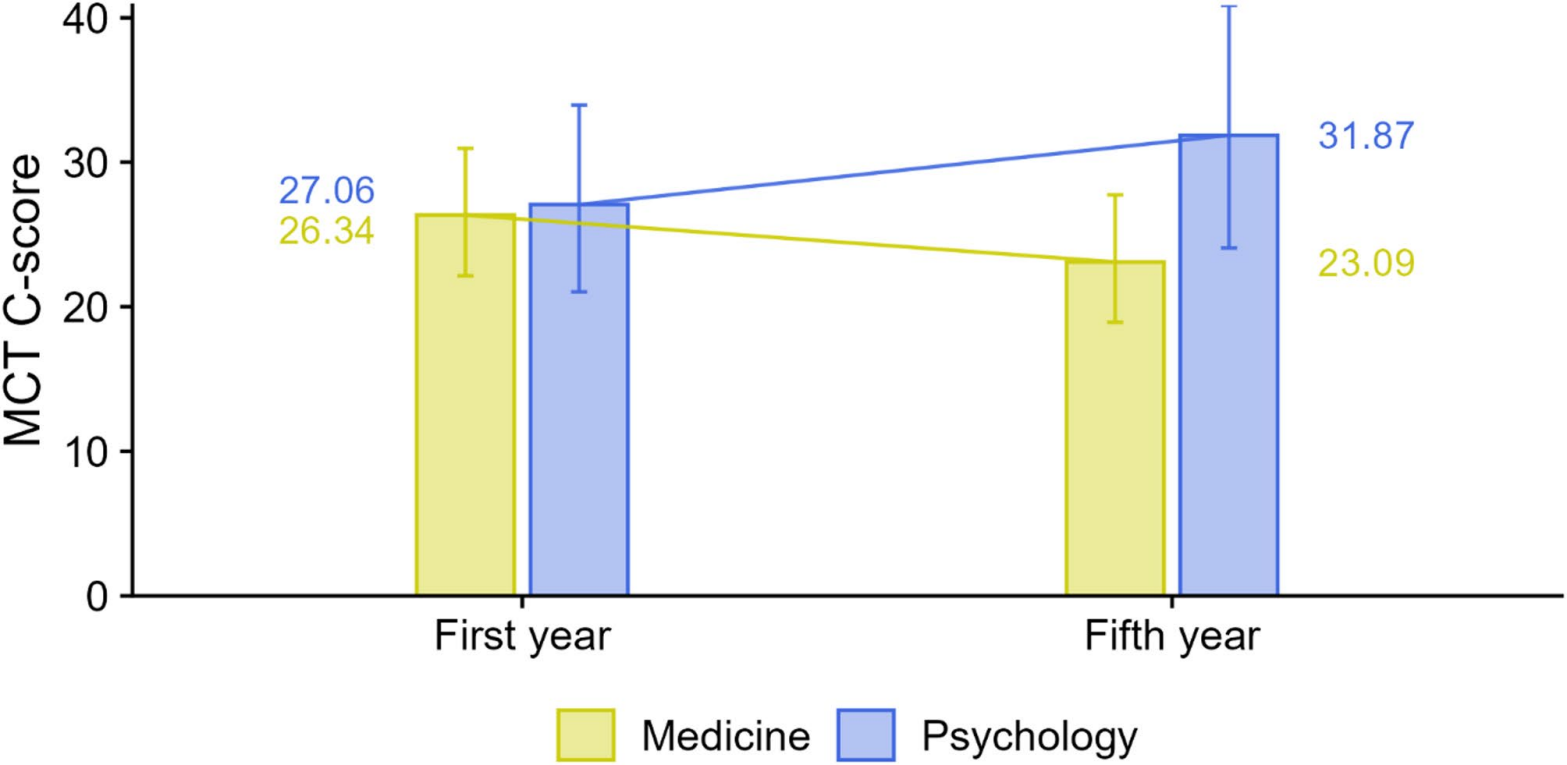
MCT C-scores among medical and psychology students by year of study: decline in medical students vs. increase in psychology students. Notes. Outcome = MCT C-score (1–100, higher = greater moral competence). Points show EMMs; whiskers = 95% CIs. Groups: Medicine vs. Psychology; First year vs. Fifth year. Sample. *n* = 717. Key tests. Field × Year interaction: *t*(708.68) = 2.23, *p* = 0.026 (significant). First year: *d* = 0.05 (ns); fifth year: Psych > Med by *d* = 0.56. Model fit. The marginal *R*^*2*^ was 0.027, indicating that the fixed effects accounted for about 3% of the variance. Abbreviations. MCT = Moral Competence Test; C-score = Moral Competence Test score (index of moral competence)

Differences between the students were also observed in the area of moral competence (RQ2). In the first year, there were virtually no differences between the fields (d = 0.05, small effect; t(6.58) = 0.22, *p* = 0.829). However, in the fifth year, psychology students exhibited C-scores approximately 0.56 standard deviations (medium effect) higher than those of medical students, t(13.55) = 2.24, *p* = 0.043. The interaction term, which indicates a differential change over time between the two fields, was statistically significant, t(708.68) = 2.23, *p* = 0.026 (see Fig. 2). Moreover, no statistically significant difference was observed between male and female participants.

Certain variables, such as test completion time and respondents’ trait anxiety, may associated with the MCT C-score (RQ3). Adding these predictors, including their interactions with the *study field* variable, significantly improved the statistical model, χ²(6) = 17.15, *p* = 0.009. Tests of individual effects confirmed a statistically significant association between completion time and moral competence among medical students, regardless of their level of trait anxiety. Specifically, medical students who took more time to complete the MCT tended to show higher levels of moral competence, indicating a correlational (not causal) association between response time and the C-score. This relationship should therefore be interpreted with caution.

**Fig. 3:**
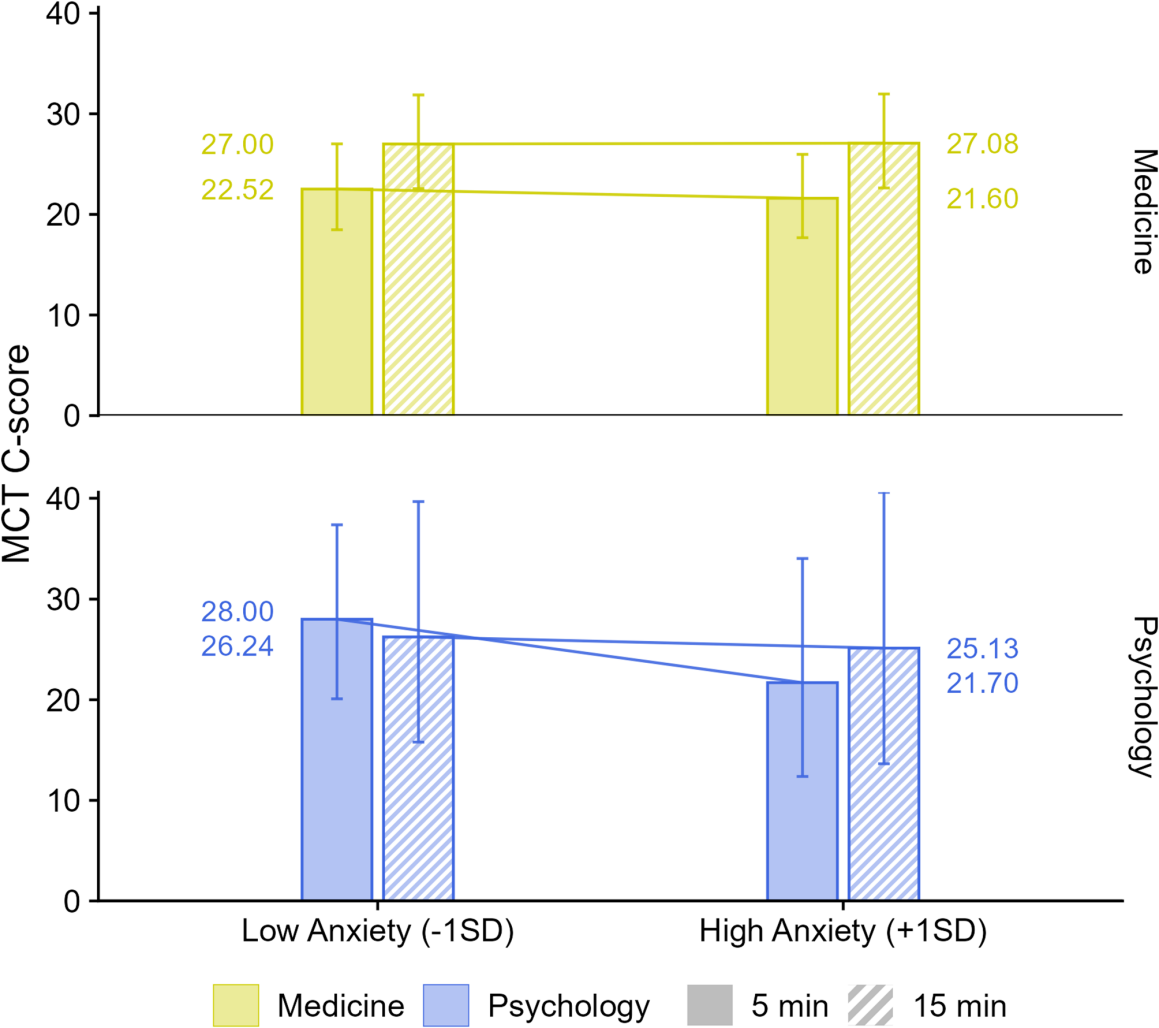
The relationship between moral competence, questionnaire completion time, and low or high levels of trait anxiety. Notes. Outcome = MCT C-score (1–100, higher = greater moral competence). Points show estimated marginal means (EMMs) across completion times from the mixed-effects model (square-root link); whiskers = 95% CIs. Groups: Medicine vs. Psychology; lower (− 1 SD) vs. higher (+ 1 SD) trait anxiety (STAI-X2). Sample. *n* = 652 (listwise deletion due to missing anxiety). Key tests. Medicine: longer completion time associated with higher C-scores (15–5 min: *d* ≈ 0.35; *t*(637.41) = 3.85, *p* < 0.001). Psychology: slope ns (*d* ≈ 0.06; *t*(635.75) = 0.17, *p* = 0.863). No evidence that trait anxiety moderated the time–C-score association. (Overall model comparison: χ²(6) = 17.15, *p* = 0.009.) Model fit. Marginal *R*^*2*^ = 0.043 (~ 4% of variance explained by fixed effects). Abbreviations. MCT = Moral Competence Test; C-score = Moral Competence Test score (index of moral competence)

The effect size of completion time among medical students can be illustrated by comparing the estimated marginal means for those who completed the test in 5 min (10th percentile) versus 15 min (90th percentile). Students in the latter group scored 0.35 standard deviations higher (small to medium effect), *t*(637.41) = 3.85, *p* < 0.001. Among psychology students, no such difference was observed, *d* = 0.06 (small effect), *t*(635.75) = 0.17, *p* = 0.863 (see Fig. 3).

**Fig. 4:**
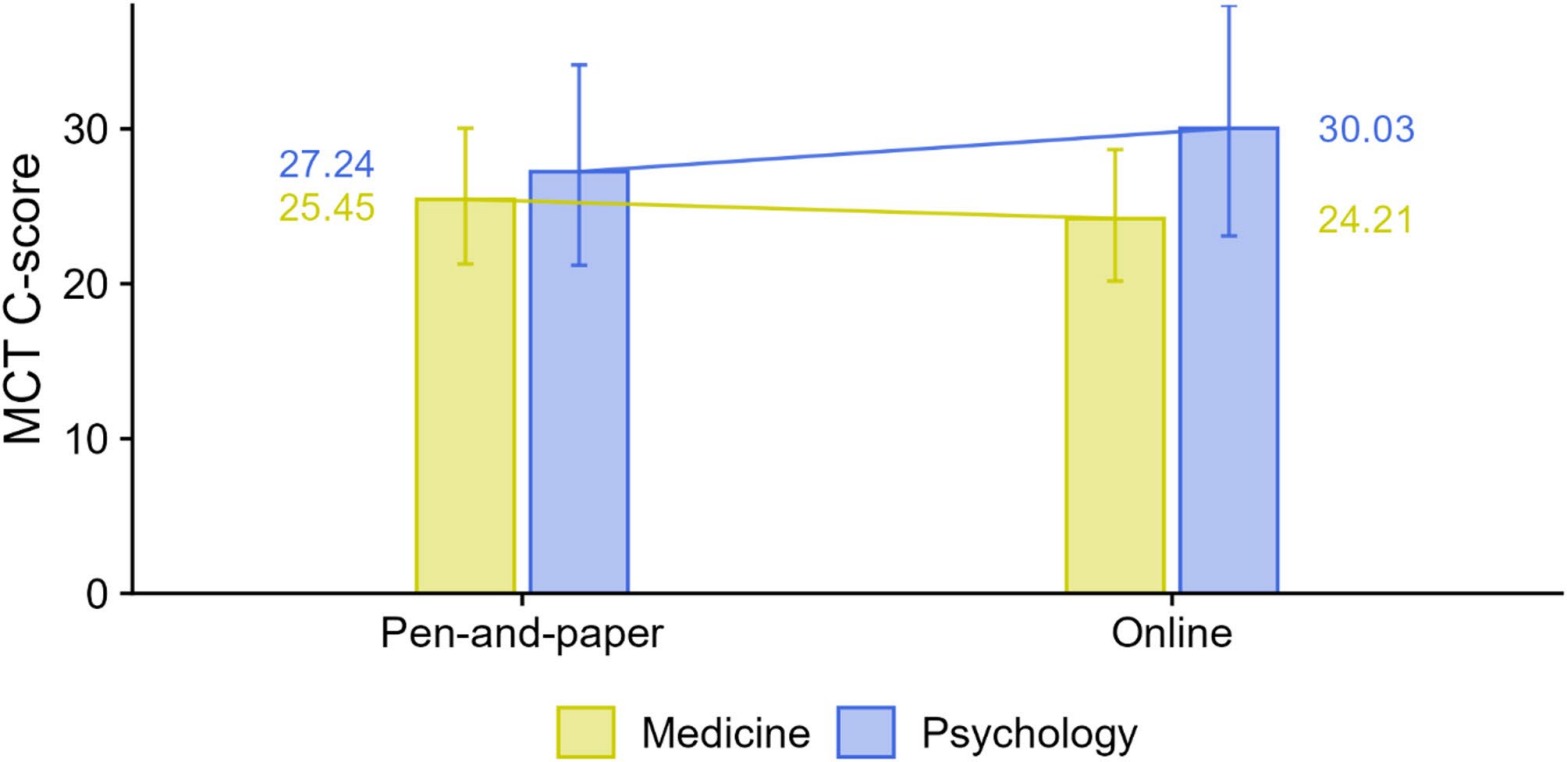
Comparison of medical and psychology students in online vs. paper-based testing. Notes. Outcome = MCT C-score (1–100). Points show EMMs; whiskers = 95% CIs. Groups split by Field (Medicine vs. Psychology) and Format (Online vs. Paper-based). Sample. Online *n* = 264, Paper-based *n* = 453 (balanced across faculties/years by systematic assignment; one site all online). Key tests. No significant format effects within either field: Psychology (Online > Paper) *d* = 0.17, *t*(708.72) = 0.83, *p* = 0.409; Medicine (Paper > Online) *d* = − 0.08, *t*(232.07) = − 0.80, *p* = 0.423. Difference in format effects between fields: *t*(670.45) = − 1.11, *p* = 0.266 (ns). Abbreviations. MCT = Moral Competence Test; C-score = index of structural consistency in moral reasoning

The results (RQ4) do not suggest an effect of the data collection method. For neither study field did we observe a statistically significant difference in MCT C-scores between tests administered online and those administered using a paper-based method. Psychology students achieved slightly higher scores in the online condition, *d* = 0.17 (small effect), *t*(708.72) = 0.83, *p* = 0.409, whereas medical students performed slightly better in the paper-based condition, *d* = −0.08 (small effect), *t*(232.07) = −0.80, *p* = 0.423. However, the difference in effect sizes was not statistically significant, *t*(670.45) = −1.11, *p* = 0.266 (see Fig. 4).

## Discussion

### Summary of findings

This study examined differences in anxiety and moral competence between medical and psychology students across two testing formats and years of study. We found that anxiety levels remained elevated among medical students, whereas they decreased among psychology students. Female students consistently reported higher anxiety than male students. Regarding moral competence, psychology students scored higher in the final year than medical students, whose scores showed a decline. Longer test completion time was positively associated with moral competence among medical students, although this relationship was not observed in psychology students. No significant differences were found between online and paper-based testing formats.

### Contextualization of anxiety and moral competence

First and foremost, it is important to contextualize the overall results presented in Table 2 in order to understand the level of anxiety experienced by medical and psychology students, as well as to interpret the development of their moral competence. The meta-analysis by Knowles and Olatunji [37] found that individuals with depressive disorders exhibit higher levels of trait anxiety than those with anxiety disorders. Moreover, the difference in trait anxiety scores between individuals with anxiety disorders and nonclinical populations was significantly greater in the United States compared to most European countries. In nonclinical populations, the average trait anxiety score is approximately 35 points. In comparison, our observed mean score of 45.41 (see Table 2) is considerably higher than the nonclinical average. However, it does not reach the levels typically reported in clinical populations. Instead, it most closely aligns with mild anxiety disorders, such as specific phobias. For reference, individuals diagnosed with Generalized Anxiety Disorder, Depression, or Post-Traumatic Stress Disorder commonly score around 60 points or higher on trait anxiety measures.

When comparing partial C-score results for the Workers’ and Doctor’s dilemma, medical students often show a marked decline in the latter. This finding is confirmed by the results of our own study (see Table 2), which also identified a decrease in C-scores specifically in response to the Doctor’s dilemma. These differences in subscores help explain why the overall C-score of medical students approaches the lower threshold, particularly in clinical years. This phenomenon, known as moral segmentation, is particularly observed among students in advanced years of medical training. Religiosity may also contribute to this effect [15]. The central issue in the Doctor’s dilemma scenario is euthanasia, which—especially in the context of preparing for the medical profession—may pose a cognitive and ethical burden that affects students’ impartial evaluation of moral arguments. For this reason, some researchers studying moral competence in medical students suggest replacing the medically focused scenario with an alternative, such as a dilemma involving a judge deciding whether to permit the torture of a suspect in a potential mass terrorist attack [38].

### The weight of the white coat: anxiety persists for medical students but fades for psychology students

Our results (RQ1) demonstrate a similar rate of trait anxiety in first-year medical and psychology students; however, this level of anxiety remained almost unchanged in the fifth year of medical students, unlike the decrease observed in psychology students. While at the beginning of their studies, sources of anxiety may be similar in both groups, in later stages they appear to diverge. For medical students, who spend the final years of their studies largely in clinical training within a hospital environment, this may be one factor associated with elevated anxiety levels. This aligns with the studies by Tian-Ci Quek et al. [7] and Lönn et al. [6] that prove anxiety can be heightened due to clinical training in final years. However, according to Wadi et al. [39] other factors may play a role in how anxiety is experienced, such as personality of the students (negative vs. positive thoughts, self-negligence vs. self-care), their academic resources (heavy curriculum vs. facilitative curricular aids), and the examiner (criticism vs. feedback, strict vs. kind approaches).

In line with many previous studies [40–42], our findings confirm significantly higher anxiety levels in women than in men, possibly because women are more likely to recognize their symptoms or more willing to report them [43, 44].

### Future doctors lose moral competence while psychology students gain it

In accordance with the proposed RQ2, the level of moral competence was comparable between first-year medical and psychology students, but in the final year, psychology students exhibited higher moral competence, while medical students demonstrated lower moral competence. Numerous studies have demonstrated a regression of moral competence in medical students between their first and fifth years [14–19], in contrast to psychology students, whose moral competence increases [17, 20, 21]. Several explanations may contribute to this phenomenon. Research suggests that medical students often begin their studies with a strong sense of idealism and a humanistic outlook, which over time might be replaced by pragmatism and a decline in initial ideals [14]. Factors such as exhaustion, stress, depressive episodes, and personal crises may also play a role [16]. Additionally, studies indicate that empathy tends to decrease while cynicism increases during the later years of medical education [22] or medical students develop emotional numbness over the course of their studies [45]. The impact of the hidden curriculum may further contribute to shaping these changes [15], particularly by contributing to medical students’ perception of epistemic invulnerability as helplessness, a closure to alternatives, and the acceptance of rigid professional boundaries lead them to perceive themselves not only as professionally inadequate but also as ethically incompetent [46]. Moreover, they quickly perceive the hierarchical structure of the healthcare environment and feel powerless to speak up, which leads to inner confusion followed by a defensive response in the form of emotional desensitization and justification [47].

So far, efforts to counter the declining trend in moral competence among medical students have been unsuccessful [19, 48, 49]. However, the small effect size in the pilot study of Friedrich and others [48] suggests that principle-based structured case discussions may improve moral competence among medical students, especially those with low and medium pre C-scores. In our study, effect sizes were generally small to medium according to Cohen’s convention, which indicates that although differences are not large, they still highlight meaningful trends in moral competence development. Instead of expanding the concept of moral competence, it seems more relevant to discuss how to counteract its decline among medical students. Strategies proposed in prior studies include principle-based structured case discussions [48], strengthening reflective practice and narrative approaches [19], and integrating ethics education more deeply into the medical curriculum [49]. Such interventions may help to sustain or even improve moral competence during medical training.

### The power of patience: longer reflection time enhances moral competence

In accordance with the proposed RQ3, the analysis showed that among medical students, longer completion times on the MCT were significantly associated with higher levels of moral competence, independent of their level of trait anxiety. This interpretation should be regarded as exploratory, since longer completion time may also reflect indecision or task difficulty rather than deeper reasoning. This positive relationship was not observed among psychology students, where completion time did not significantly predict moral competence.

Intuitionist and cognitivist theories of morality differ in their emphasis on whether emotions [50] or cognition [51] play a more important role for moral judgment. The intuitionist approach, through time manipulation, mainly demonstrates that time pressure creates an automated, quick response without cognitive control. The dual-process model of moral judgments manifests in moral dilemmas either faster in deontological gut reactions or slower in terms of cognitive control associated with consequentialist responses. When there is insufficient time for cognitive control, the likelihood of deontological response increases. Time (or lack thereof) acts as a gatekeeper that determines whether cognitive processes related to moral judgment can occur, regardless of whether they happen consciously or unconsciously [52, 53]. Moreover, time pressure appears to be a very important factor that weakens the moral awareness of the individual and can even lead to inappropriate or abusive behavior [54]. Our findings suggest, in line with prior research, that sufficient reflection time may facilitate consistent moral reasoning, but causality cannot be inferred.

### Online or paper-based? Moral competence remains unchanged

With regard to RQ4, our findings revealed no statistically significant differences in the level of moral competence between participants who completed the questionnaires online and those who completed them in a traditional paper-based format. Nonetheless, prior research indicates that the medium of communication may influence ethical behavior. For example, Naquin et al. [55] found that individuals tend to lie more frequently in email communication compared to handwritten formats and also feel more justified in doing so. Furthermore, behavioral manifestations of moral development have been documented in classic experimental settings. In a study by Krebs and Rosenwald [56], participants who had completed Kohlberg’s Test of Moral Development were asked to return additional personality questionnaires by mail. Those with higher levels of moral development were significantly more likely to return the materials on time, while those with moderate scores returned them after the deadline, and individuals with the lowest scores failed to return them at all. These findings suggest that although the mode of test administration may not influence measured moral competence directly, it could be associated with differences in behavior that reflect underlying moral traits, particularly in real-world or follow-up situations.

### Educational implications

Reducing or preventing anxiety in medical students can be facilitated through targeted interventions, including stress management programs, resilience training, and mindfulness techniques [57, 58]. As Slavin et al. [59] shows, curricular changes - such as adjustments in course content, scheduling, or grading - can significantly lower depression and anxiety levels as well.

Despite all the mentioned factors contributing to the decline in moral competence, in the context of Czech educational practice in medical faculties, a possible explanation may lie in the limited influence of ethics education. Czech curricula tend to teach principle-based ethics, mainly through frontal instruction or discussions on ethical case studies; however, up-to-date pedagogical approaches that influence students’ moral development, such as reflective practice, narrative medicine, or role-playing including the use of VR, remain rarely used. Additionally, little focus is given to how teachers, as role models, influence students’ moral and professional development.

Moreover, addressing hidden curricula by developing institutional strategies for educating teachers and clinical mentors—who serve as role models for students—would be beneficial. Cultivating an ethical academic and clinical environment could further support students in developing their moral competence. Since medical students experience persistent anxiety at higher rates than their peers in other disciplines, universities should encourage interdisciplinary interactions. Opportunities for informal or extracurricular activities, such as sports or collaborative events, could help alleviate stress and foster peer support. Additionally, students in healthcare-related fields, including medicine, nursing, and clinical psychology, could benefit from joint ethics courses or moral competence seminars, where they can engage in discussions and share diverse perspectives on ethical dilemmas.

Given that clinical training presents students with high-pressure situations requiring immediate ethical decision-making, simulation-based ethics training—through role-playing or VR—could offer a valuable tool for preparation. Such methods would allow students to experience high-stress ethical scenarios in a controlled, low-risk environment, with the added benefit of structured debriefing before encountering similar challenges in real clinical practice. By implementing these strategies, medical faculties can contribute not only to reducing student anxiety but also to strengthening their moral competence and professional integrity.

### Limitations and future directions

This study has several limitations. First, it was challenging to ensure an even distribution of first- and fifth-year students across institutions and testing formats. At one site, all students were required to participate online, and in the case of psychology students, it was difficult to identify suitable fifth-year courses for testing. Second, self-reported anxiety may not fully capture physiological or behavioral aspects of anxiety. Third, the assessment of moral competence relied on the MCT, which focuses primarily on moral judgment and does not include other relevant aspects such as sensitivity, motivation, or behavior. Fourth, because the study design was cross-sectional, it cannot capture developmental trajectories. Finally, the online format may have introduced variability in participant attentiveness, and individual differences in test anxiety could not be systematically compared between modalities.

Although the study has certain methodological constraints, its findings highlight relevant trends with implications for medical and psychology education. Future research may explore interventions aimed at reducing anxiety while fostering moral competence in medical education. The role of gender in anxiety perception and reporting deserves further investigation, as this may inform the design of more effective mental health interventions. Longitudinal designs are needed to track changes in anxiety and moral competence over time and into professional practice. Innovative training approaches, such as simulation-based or virtual reality methods, may offer promising ways to prepare students for ethically challenging clinical situations. Additionally, future work should aim to develop new instruments that capture not only moral judgment but also broader abilities relevant to moral functioning.

## Conclusion

Moral competence and anxiety levels were found to be comparable in first-year medical and psychology students. However, in the final year, psychology students exhibited higher moral competence and lower anxiety, whereas medical students demonstrated lower moral competence, with anxiety levels remaining consistent with those observed in the first year. Female students consistently self-reported significantly higher levels of trait anxiety compared to male students. Medical students who took more time to complete the Moral Competence Test tended to show higher levels of moral competence, although this association should be interpreted cautiously and does not imply causality. In contrast, no such effect was found among psychology students. No significant differences in moral competence were observed between respondents who completed the questionnaires online versus on paper, and the generalizability of these findings is limited by the cultural and curricular specificities of Czech institutions, the cross-sectional design, and the reliance on self-report measures of anxiety.

## Data Availability

The datasets used and/or analyzed during the current study are available from the corresponding author upon reasonable request.

## Abbreviations

C-score: Moral Competence Test score (index of moral competence)
EMMs: Estimated Marginal Means
MCT: Moral Competence Test
RQ: Research Question
SD: Standard Deviations
STAI: State–Trait Anxiety Inventory
X1: State Anxiety subscale of STAI
X2: Trait Anxiety subscale of STAI

## References

[1] Dyrbye LN, Thomas MR, Shanafelt TD. Systematic review of depression, anxiety, and other indicators of psychological distress among US and Canadian medical students. Acad Med. 2006;81(4):354–73. 16565188 10.1097/00001888-200604000-00009

[2] Barlow DH. Anxiety and its disorders: the nature and treatment of anxiety and panic. New York: Guilford Press; 2004.

[3] Spielberger CD. Theory and research on anxiety. Anxiety and behavior. New York: Academic; 1966.

[4] Dahlin M, Joneborg N, Runeson B. Stress and depression among medical students: a cross-sectional study. Med Educ. 2005;39(6):594–604. 15910436 10.1111/j.1365-2929.2005.02176.x

[5] Heinen I, Bullinger M, Kocalevent R-D. Perceived stress in first year medical students-associations with personal resources and emotional distress. BMC Med Educ. 2017;17:1–14. 28056972 10.1186/s12909-016-0841-8 PMC5216588

[6] Lönn A, Weurlander M, Seeberger A, Hult H, Thornberg R, Wernerson A. The impact of emotionally challenging situations on medical students’ professional identity formation. Adv Health Sci Educ Theory Pract. 2023;28(5):1557–78. 37184676 10.1007/s10459-023-10229-8 PMC10184105

[7] Tian-Ci Quek T, Wai-San Tam W, Tran X, Zhang B, Zhang M, Su-Hui Ho Z, Chun-Man Ho C. The global prevalence of anxiety among medical students: a meta-analysis. Int J Environ Res Public Health. 2019;16(15):2735. 31370266 10.3390/ijerph16152735 PMC6696211

[8] Dyrbye LN, Thomas MR, Shanafelt TD. Medical student distress: causes, consequences, and proposed solutions. Mayo Clin Proc. 2005;80(12):1613–22. 16342655 10.4065/80.12.1613

[9] Sarikaya O, Civaner M, Kalaca S. The anxieties of medical students related to clinical training. Int J Clin Pract. 2006;60(11):1414–8. 16787438 10.1111/j.1742-1241.2006.00869.x

[10] Voltmer E, Köslich-Strumann S, Voltmer J-B, Kötter T. Stress and behavior patterns throughout medical education–a six year longitudinal study. BMC Med Educ. 2021;21:1–12. 34454487 10.1186/s12909-021-02862-x PMC8403353

[11] González Ramírez MT, Landero Hernández R, García-Campayo J. Relación Entre La depresión, La Ansiedad y Los síntomas psicosomáticos En Una muestra de estudiantes universitarios Del Norte de México. Rev Panam Salud Publica. 2009;25(2):141–5. 19531309 10.1590/s1020-49892009000200007

[12] Lima Junior AMd França, GHBd, Oltramari G, Andrade ALM, Lopes FM. Saúde mental de profissionais e estudantes de psicologia brasileiros: Uma Análise comparativa. Paideia (Ribeirao Preto). 2025;34:e3433.

[13] Lind G. How to teach moral competence. Berlin: Logos Verlag Berlin GmbH; 2019.

[14] Hegazi I, Wilson I. Medical education and moral segmentation in medical students. Med Educ. 2013;47(10):1022–8. 24016172 10.1111/medu.12252

[15] Neves Feitosa H, Rego S, Unger Raphael Bataglia P, Castelo Branco Sancho KF, Rego G, Nunes R. Moral judgment competence of medical students: a transcultural study. Adv Health Sci Educ Theory Pract. 2013;18:1067–85. 23463178 10.1007/s10459-013-9449-5

[16] Nowak E, Barciszewska A-M, Hemmerling K, Lind G, Taradi SK. Giving moral competence high priority in medical education. New MCT-based research findings from the Polish context. ETHICS IN PROGRESS. 2021;12(1):104–33.

[17] Schillinger M. Learning environment and moral development: how university education fosters moral judgment competence in Brazil and two German-speaking countries. Aachen: Shaker; 2006.

[18] Slováčková B, Slováček L. Moral judgement competence and moral attitudes of medical students. Nurs Ethics. 2007;14(3):320–8. 17459816 10.1177/0969733007075867

[19] Zielina M, Škoda J, Ivanová K, Dostál D, Juríčková L, Anthony Procházka D, et al. Exploring moral competence regression: a narrative approach in medical ethics education for medical students. BMC Med Ethics. 2024;25(1):73. 38907238 10.1186/s12910-024-01073-5 PMC11191321

[20] Chvojková P. Interkulturní aspekty morální kompetence. (Master’s thesis). Brno: Masarykova univerzita; 2013.

[21] Lind G. Empirical findings on the cross-cultural validity of the Moral Judgment Test (MJT). Paper presented at: Annual Meeting of the American Educational Research Association. Chicago, IL. 2003.

[22] Self DJ, Schrader D, Baldwin D Jr, Wolinsky F. The moral development of medical students: a pilot study of the possible influence of medical education. Med Educ. 1993;27(1):26–34. 8433656 10.1111/j.1365-2923.1993.tb00225.x

[23] Olson LM. The relationship between moral integrity, psychological well-being, and anxiety. Doctoral dissertation. University of Wisconsin–Madison, Madison (WI); 2002. [dissertation]. Madison (WI): University of Wisconsin–Madison; 2002.

[24] Kouchaki M, Desai SD. Anxious, threatened, and also unethical: how anxiety makes individuals feel threatened and commit unethical acts. J Appl Psychol. 2015;100(2):360. 25243997 10.1037/a0037796

[25] Zhao J, Harris M, Vigo R. Anxiety and moral judgment: the shared deontological tendency of the behavioral inhibition system and the unique utilitarian tendency of trait anxiety. Pers Individ Dif. 2016;95:29–33.

[26] Hashimoto H, Maeda K, Matsumura K. Fickle judgments in moral dilemmas: time pressure and utilitarian judgments in an interdependent culture. Front Psychol. 2022;13:795732. 35310271 10.3389/fpsyg.2022.795732 PMC8928142

[27] Del Popolo Cristaldi F, Palmiotti GP, Cellini N, Sarlo M. Pulling the lever in a hurry: the influence of impulsivity and sensitivity to reward on moral decision-making under time pressure. BMC Psychol. 2024;12(1):270. 38745341 10.1186/s40359-024-01773-y PMC11092183

[28] Tinghög G, Andersson D, Bonn C, Johannesson M, Kirchler M, Koppel L, et al. Intuition and moral decision-making–the effect of time pressure and cognitive load on moral judgment and altruistic behavior. PLoS ONE. 2016;11(10):e0164012. 27783704 10.1371/journal.pone.0164012 PMC5082681

[29] Alruwais N, Wills G, Wald M. Advantages and challenges of using e-assessment. Int J Inf Educ Technol. 2018;8(1):34–7.

[30] Cassady JC, Gridley BE. The effects of online formative and summative assessment on test anxiety and performance. J Technol Learn Assess. 2005;4(1):1–31.

[31] Ewell SN, Josefson CC, Ballen CJ. Why did students report lower test anxiety during the COVID-19 pandemic? J Microbiol Biol Educ. 2022;23(1):e00282–00221. 35496685 10.1128/jmbe.00282-21 PMC9053057

[32] Solati A, Amani A, Armat MR. Impact of learning environment on reading anxiety: a study of medical students in online and traditional settings. BMC Med Educ. 2024;24(1):1502. 39707435 10.1186/s12909-024-06516-6 PMC11660883

[33] Sahlan A, Madil W, Hutnisyawati. The effects of modes of test administration on test anxiety and test scores: A study in an Indonesian school. Issues Educ Res. 2021;31(3):952–71.

[34] Classen B. Online/Offline discrepancies in moral judgement among social media users: A mixed methods study. Doctoral dissertation. [PhD thesis]. Wellington: Victoria University of Wellington; 2024.

[35] Hedges LV. Effect sizes in cluster-randomized designs. J Educ Behav Stat. 2007;32(4):341–70.

[36] Bates D, Mächler M, Bolker BM, Walker SC. Fitting linear mixed-effects models using lme4. J Stat Softw. 2015;67(1):1–48.

[37] Knowles KA, Olatunji BO. Specificity of trait anxiety in anxiety and depression: meta-analysis of the state-trait anxiety inventory. Clin Psychol Rev. 2020;82:101928. 33091745 10.1016/j.cpr.2020.101928 PMC7680410

[38] Martins VSM, Santos CMNC, Bataglia PUR, Duarte IMRF. The teaching of ethics and the moral competence of medical and nursing students. Health Care Anal. 2021;29:113–26. 32944887 10.1007/s10728-020-00401-1 PMC8106588

[39] Wadi M, Yusoff MSB, Abdul Rahim AF, Lah NAZN. Factors affecting test anxiety: a qualitative analysis of medical students’ views. BMC Psychol. 2022;10(1):8. 34991718 10.1186/s40359-021-00715-2 PMC8739979

[40] Brenneisen Mayer F, Souza Santos I, Silveira PS, Itaqui Lopes MH, de Souza ARND, Campos EP, de Abreu BAL, Hoffman II, Magalhães I, Lima CR. Factors associated to depression and anxiety in medical students: a multicenter study. BMC Med Educ. 2016;16:1–9. 27784316 10.1186/s12909-016-0791-1 PMC5080800

[41] Cipra C, Müller-Hilke B. Testing anxiety in undergraduate medical students and its correlation with different learning approaches. PLoS ONE. 2019;14(3):e0210130. 30865635 10.1371/journal.pone.0210130 PMC6415780

[42] Memon I, Omair A, Barradah OM, Almegren NM, Almuqbil MM, Batarfi OH, Masuadi E, Feroz Z, OMAIR, Barradah DA. O. Measurement of exam anxiety levels among medical students and their association with the influencing factors. Cureus 2023;15(7):e41417. 10.7759/cureus.41417 PMC10403227 37546066

[43] McLean CP, Asnaani A, Litz BT, Hofmann SG. Gender differences in anxiety disorders: prevalence, course of illness, comorbidity and burden of illness. J Psychiatr Res. 2011;45(8):1027–35. 21439576 10.1016/j.jpsychires.2011.03.006 PMC3135672

[44] Núñez-Peña MI, Suárez-Pellicioni M, Bono R. Gender differences in test anxiety and their impact on higher education students’ academic achievement. Procedia. 2016;228:154–60.

[45] Pinkasová T, Fialová L. I began to Wonder whether I am becoming emotionally numb. Sociocultural background, hidden curriculum, and moral self-reflection in the development of medical professional identity: A qualitative study. Rev Esp Pedagog. 2025;83(291):16.

[46] Hafferty FW, Franks R. The hidden curriculum, ethics teaching, and the structure of medical education. Acad Med. 1994;69(11):861–71. 7945681 10.1097/00001888-199411000-00001

[47] McDonald J, Graves J, Abrahams N, Thorneycroft R, Hegazi I. Moral judgement development during medical student clinical training. BMC Med Educ. 2021;21:1–9. 33653350 10.1186/s12909-021-02572-4 PMC7927259

[48] Friedrich O, Hemmerling K, Kuehlmeyer K, Nörtemann S, Fischer M, Marckmann G. Principle-based structured case discussions: do they foster moral competence in medical students?-A pilot study. BMC Med Ethics. 2017;18:1–8. 28253882 10.1186/s12910-017-0181-1 PMC5335793

[49] Nadolny S, Bruns F, Nowak A, Schildmann J. Moral competency of students at a German medical school–a longitudinal survey. BMC Med Educ. 2024;24(1):691. 38918781 10.1186/s12909-024-05674-x PMC11201357

[50] Haidt J. The emotional dog and its rational tail: a social intuitionist approach to moral judgment. Psychol Rev. 2001;108(4):814. 11699120 10.1037/0033-295x.108.4.814

[51] Kohlberg L. The philosophy of moral development: moral stages and the Idea of justice. New York: Harper & Row; 1981.

[52] Greene JD, Morelli SA, Lowenberg K, Nystrom LE, Cohen JD. Cognitive load selectively interferes with utilitarian moral judgment. Cognition. 2008;107(3):1144–54. 18158145 10.1016/j.cognition.2007.11.004 PMC2429958

[53] Suter RS, Hertwig R. Time and moral judgment. Cognition. 2011;119(3):454–8. 21354557 10.1016/j.cognition.2011.01.018

[54] Zhang Z, Jia X. No time for ethics: how and when time pressure leads to abusive supervisory behavior. J Bus Ethics. 2023;188(4):807–25.

[55] Naquin CE, Kurtzberg TR, Belkin LY. The finer points of lying online: e-mail versus pen and paper. J Appl Psychol. 2010;95(2):387. 20230078 10.1037/a0018627

[56] Krebs D, Rosenwald A. Moral reasoning and moral behavior in conventional adults. Merrill Palmer Q Behav Dev. 1977;23(2):77–87.

[57] Fino E, Martoni M, Russo P. Specific mindfulness traits protect against negative effects of trait anxiety on medical student wellbeing during high-pressure periods. Adv Health Sci Educ Theory Pract. 2021;26(3):1095–111. 33675487 10.1007/s10459-021-10039-w PMC8338863

[58] Shao R, He P, Ling B, Tan L, Xu L, Hou Y, Kong L, Yang Y. Prevalence of depression and anxiety and correlations between depression, anxiety, family functioning, social support and coping styles among Chinese medical students. BMC Psychol. 2020;8:1–19. 32321593 10.1186/s40359-020-00402-8 PMC7178943

[59] Slavin SJ, Schindler DL, Chibnall JT. Medical student mental health 3.0: improving student wellness through curricular changes. Acad Med. 2014;89(4):573–7. 24556765 10.1097/ACM.0000000000000166 PMC4885556

